# RaSS: 4D mm-Wave Radar Point Cloud Semantic Segmentation with Cross-Modal Knowledge Distillation

**DOI:** 10.3390/s25175345

**Published:** 2025-08-28

**Authors:** Chenwei Zhang, Zhiyu Xiang, Ruoyu Xu, Hangguan Shan, Xijun Zhao, Ruina Dang

**Affiliations:** 1 College of Information Science and Electronic Engineering, Zhejiang University, Hangzhou 310027, China; zhangchenwei@zju.edu.cn (C.Z.); xuruoyu@zju.edu.cn (R.X.); hshan@zju.edu.cn (H.S.); 2 Zhejiang Provincial Key Laboratory of Mutil-Modal Communication Networks and Intelligent Information Processing, Zhejiang University, Hangzhou 310027, China; 3 ChinaNorth Artificial Intelligence & Innovation Research Institute, Beijing 100072, China; heejunzhao@163.com (X.Z.); ruinadang@163.com (R.D.)

**Keywords:** radar, semantic segmentation, knowledge distillation

## Abstract

Environmental perception is an essential task for autonomous driving, which is typically based on LiDAR or camera sensors. In recent years, 4D mm-Wave radar, which acquires 3D point cloud together with point-wise Doppler velocities, has drawn substantial attention owing to its robust performance under adverse weather conditions. Nonetheless, due to the high sparsity and substantial noise inherent in radar measurements, most radar perception studies are limited to object-level tasks, with point-level tasks such as semantic segmentation remaining largely underexplored. This paper aims to explore the possibility of using 4D radar in semantic segmentation. We set up the ZJUSSet dataset containing accurate point-wise class labels for radar and LiDAR. Then we propose a cross-modal distillation framework RaSS to fulfill the task. An adaptive Doppler compensation module is also designed to facilitate the segmentation. Experimental results on ZJUSSet and VoD dataset demonstrate that our RaSS model significantly outperforms the baselines and competitors. Code and dataset will be available upon paper acceptance.

## 1. Introduction

Semantic segmentation stands as a critical task in the field of autonomous driving. Currently, semantic segmentation techniques relying on images or LiDAR point cloud have undergone extensive research and reached a mature stage. Despite significant advancements, image or LiDAR-based segmentation remains challenged under adverse illumination or weather conditions. By contrast, mm-Wave radar can operate under adverse weathers such as snow and fog, and acquire point cloud with Doppler velocities, which makes them a popular sensor for environmental perception.

Based on the dimensionality of the point cloud, mm-Wave radar can be categorized into 3D and 4D. The former produces 2D point cloud while the latter can produce 3D point cloud similar to 3D LiDAR. However, in contrast to LiDAR, the point cloud generated by 4D radar is extremely sparse, boasting a density merely one-tenth that of LiDAR. Furthermore, owing to the multi-path effect intrinsic to millimeter waves, the range measurements of radar are substantially noisier than those of LiDAR. Therefore, most studies use radar as an auxiliary sensor and focus on fusing radar with other modalities [1,2].

Majority of current 4D radar studies focus on object-level tasks [3,4], prioritizing object presence and position with bounding boxes. Point-level semantic segmentation, by contrast, labels each point to enable detailed environmental understanding that encompasses objects and backgrounds. This is critical for fine-grained autonomous driving. As a result, current mm-Wave radar datasets primarily focus on 2D or 3D object detection tasks [5,6]. A very small number of them are involved in semantic segmentation task [7,8,9], with only 2D point cloud provided. The lack of 4D radar-based semantic segmentation datasets hinders further study and exploration of this point-based classification problem. In addition, the sparse points and large noise contained in the 4D radar data also call for novel methods for semantic segmentation.

**Figure 1:**
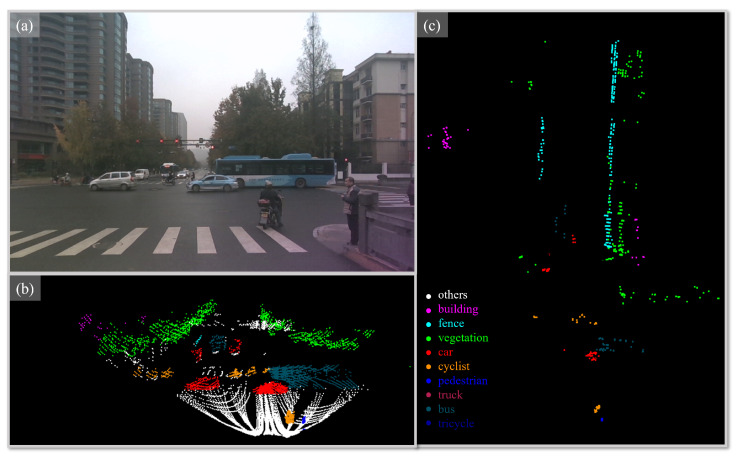
A data example of the ZJUSSet. The top-left panel (**a**) displays the camera image. The bottom left (**b**) and right (**c**) show the corresponding 128-line Livox LiDAR and Oculii 4D radar point cloud, with annotated categories marked by different colors.

To tackle these challenges, this paper proposes a new semantic segmentation dataset ZJUSSet, which comprises synchronized 4D mm-Wave radar, LiDAR, and camera data acquired in urban environments. The sensors are carefully calibrated to produce spatial aligned data. Up to 10 categories are annotated in order to provide a comprehensive description of the scene. Several sample cases from the dataset are illustrated in Figure 1.

Building on the ZJUSSet, we propose RaSS, a cross-modal distillation-based semantic segmentation framework, to tackle the challenges stemming from the marked sparsity and noise present in radar point cloud. The framework employs a pre-trained LiDAR-based segmentation model in the role of teacher, with the radar-only model serving as its student counterpart. With much higher density and accuracy, the LiDAR-based teacher can produce features containing rich structural and semantic information for the segmentation task. Knowledge distillation guides radar to learn strong features analogous to LiDAR’s, thereby improving the feature representation of the Student model. In addition, an Adaptive Doppler Compensation (ADC) module is designed to strengthen the distinguishing capability of static and non-static objects. Results from experiments on ZJUSSet demonstrate that our RaSS model not only yields a 5.57% mIOU improvement over the baseline but also outperforms existing methods with superior performance.

The main contributions are summarized as follows:

- We present ZJUSSet, a new dataset designed specifically for 4D radar semantic segmentation. To our knowledge, it is the first dataset offering point-level annotations of 4D radar across 10 categories.
- We develop a cross-modal knowledge distillation framework for semantic segmentation of 4D radar point cloud. Leveraging spatially aligned feature maps from a LiDAR-based teacher model, the radar-only student model is able to learn and extract more discriminative features optimized for segmentation tasks.
- We evaluate our RaSS framework alongside other state-of-the-art approaches on both ZJUSSet and VoD [6]. The findings confirm that our model outperforms competing methods and highlight the promising prospects of 4D radar-based semantic segmentation.

## 2. Related Work

### 2.1. Radar Segmentation Dataset

Existing mm-Wave radar datasets typically include radar and camera images, most of which are designed for the task of object detection [5,6]. Merely a handful of these apply to the task of semantic segmentation on radar.

CARRADA [7] contains synchronized RGB images and 3D radar frames, enabling 2D semantic segmentation via range-Doppler or range-angle representations. In addition to object bounding box annotation, RADIal [8] also provides free-space annotations on the range-Doppler spectrum. RadarScenes [9], a large-scale multi-sensor dataset with 2D point-wise annotations, supports tasks such as object detection, clustering, classification, and semantic segmentation.

Table 1 summarizes the differences between the existing radar segmentation dataset and ours. Our ZJUSSet includes rich data from 4D radar, dense LiDAR, and camera, providing more categories with 3D point-wise annotations, which is more challenging for the segmentation task.

### 2.2. Radar-Based Semantic Segmentation Methods

Existing radar semantic segmentation methods all focus on 3D radars. According to the form of radar data used, they can be grouped into two types: tensor-based and point-based. The tensor-based methods take the radar RD tensor [10], RA tensor [11], or RAD tensor [8] as the input and segment them into different semantic regions. The point-based methods are more popular since they directly carry out segmentation on the point cloud, which is more efficient and intuitive. Furthermore, these methods can draw upon proven algorithms for LiDAR processing, eliminating the need to develop them entirely from the scratch. According to the feature presentation used, these techniques can be further partitioned into point-based [12,13] and grid-based methodologies [14,15].

With a radar point cloud as input, Ole [16] employed PointNet [17] as the feature extractor. Nobis [13] introduced a KPConv [18] based long short-term memory (LSTM) to leverage the in-context formation in the radar sequence. Jakob [19] divided the radar point cloud into grids and classified the points contained in them. More recently, Gaussian Radar Transformer (GRT) [20] proposes a self-attention method for radar points, which achieves better 2D point segmentation performance on the sparse radar point cloud.

Existing algorithms, however, are confined to 3D mm-Wave radar and can only handle 2D semantic segmentation tasks.

### 2.3. Knowledge Distillation

Knowledge distillation usually refers to training a compact, low-complexity, and inference-deployment-friendly student network by introducing a complex and high-precision teacher network with knowledge transfer [21]. The student network can acquire implicit knowledge from the teacher network by different levels of distillation, such as response level [21], feature level [22], and relation level [23].

In recent years, knowledge distillation has become popular in 3D LiDAR point semantic segmentation. They are manifested primarily in feature learning and fusion [24,25], multi-modal learning [26,27], and weakly or unsupervised learning [28]. Jiang [25] distills 3D voxel characteristics onto BEV characteristics, allowing the BEV model to perceive more geometric information. Hou [24] proposes point and voxel affinity distillation to assist the student model in better capturing the features of the structure. Yan [26] extracts rich semantic features from a multi-modal teacher (camera and LiDAR) and employs cross-modal distillation to strengthen the pure LiDAR-based student networks. Furthermore, Zhang [27] utilizes foundation models such as SAM [29] and CLIP [30] to obtain dense pseudo-labels for training.

Compared to the existing cross-modal methods, our study focuses on distillation dense Lidar-based teacher to highly sparse and noisy radar-input student and exploring its potential in semantic segmentation.

## 3. 4D Radar Semantic Segmentation Dataset

**Figure 2:**
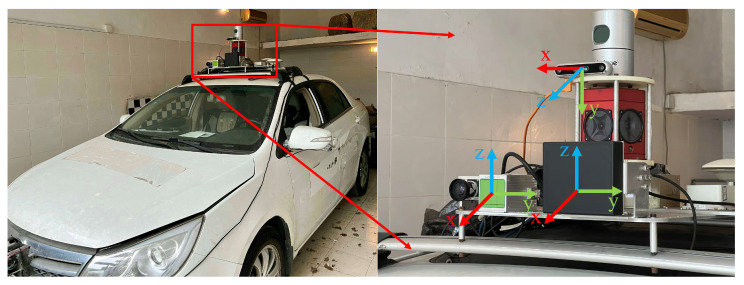
The experimental car with sensors and the coordinate system. The right image shows the enlarged view of the framed area in the left figure, with the details of the mounting sensors and their coordinate systems.

The ZJUSSet dataset was collected in Hangzhou city, China, with an experimental car platform configured with multiple sensors, as shown in Figure 2. This section will introduce the sensor setup, calibration, data annotation, and statistical information of the new dataset.

### 3.1. Sensor Setup

ZJUSSet contains data acquired from an Oculii 4D mm-Wave radar, a Livox Avia LiDAR, and a Realsense Camera. The radar supports a 400 m detection range, achieving 0.86 m distance resolution and 0.27 m/s velocity resolution, respectively. The radar boasts a field of view (FOV) measuring 113° horizontally by 45° vertically. As for the LiDAR, its FOV spans 70.4° (horizontal) by 77.2° (vertical) and the maximum detection distance of 450 m with a measurement accuracy of 2 cm. The resolution of the image is 640 × 480. All data were acquired at a synchronized frame rate of 10 Hz.

### 3.2. Calibration

Accurate calibration of the sensors is crucial. We first use a checkerboard for the intrinsic calibration of the camera, and the joint calibration between the LiDAR and the camera [31]. Since the mmw-radar and LiDAR themselves are pre-calibrated at the factory, only the extrinsic calibrations among them are required. We employ corner reflectors for radar-LiDAR and radar-camera calibration. We position corner reflectors at various locations in front of the sensors. The radar points with the highest Signal-to-Noise Ratio (SNR) near each reflector are selected to determine their positions in the radar coordinate system. Corresponding reflector positions are also identified in the LiDAR and camera data. Using the Iterative Closest Point (ICP) algorithm [32] and the Perspective-n-Point (PnP) method [33], we compute the coordinate transformations for radar-LiDAR and radar-camera pairs, respectively.

### 3.3. Data Annotation

Compared to LiDAR, the point cloud mm-Wave radar is extremely sparse, making direct annotation challenging and unreliable. Therefore, we facilitate the radar point annotation work by projecting LiDAR points onto the radar coordinates, and refer to the denser LiDAR point for annotation guidance. With the help of the image and the long-range Livox LiDAR point cloud, we can achieve precise radar point annotations within 200 m.

To relieve the labor of labeling, we first annotate the foreground objects with 3D bounding boxes. All radar and LiDAR points falling within the identical bounding box are subsequently given the same class label as the box itself. At last, we manually annotate points of the background categories such as buildings, vegetation, and fences.

Finally, the dataset encompasses ten categories: Building, Vegetation, Fence, Car, Cyclist, Pedestrian, Truck, Bus, Tricycle, and Others. Each radar point is an 8-vector, composed of coordinates (x,y,z), range *r*, the Doppler velocity *v*, the signal to noise ratio snr, Azimuth Angle α, and Elevation Angle β.

### 3.4. Statistics Analysis

In this work, we assemble the dataset with 190 sequences, each consisting of about 100 frames captured at 10 Hz. Annotations were made every 5 frames, resulting in a total of 3794 annotated frames within 19,000 frames of raw data. The initial 3000 annotated frames serve as the training set, while the rest 794 annotation frames are reserved for validation purpose.

**Figure 3:**
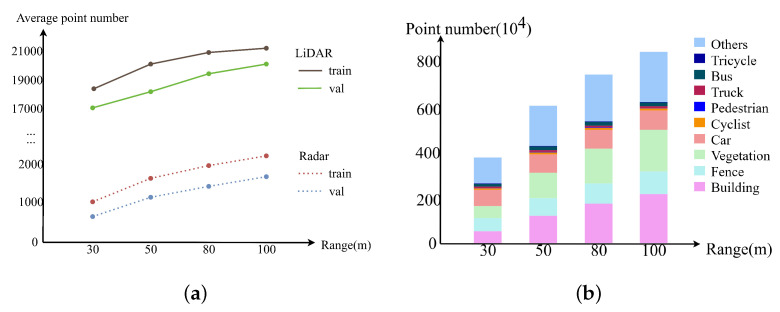
Distribution of radar points on ZJUSSet. (**a**) shows the average point number per frame for the data splits with respect to the range, while (**b**) shows the point distribution of each category in the entire train+val set.

The distribution of the radar point is shown in Figure 3, where we can see that the average quantity of radar point is less than one-tenth of the LiDAR. In the meantime, the point distributions among the categories are not uniform, with much fewer points in the categories of Pedestrian, Truck, Bus, and Tricycle. All such aspects underscore the distinct characteristic of radar points and highlight the unique challenges in 4D radar semantic segmentation task.

## 4. Method

### 4.1. Framework

The primary objective of this study is to investigate radar point cloud for the semantic segmentation task and set up a benchmark for the ZJUSSet. As there is no similar work previously, we employ 3D semantic segmentation network that is originally designed for LiDAR as the baseline for our 4D radar-based segmentation task. To tackle the emerging challenges arising from the high sparsity and noise in 4D radar point cloud, we put forward a cross-modal knowledge distillation-based Radar Semantic Segmentation (RaSS) framework aimed at boosting model performance.

**Figure 4:**
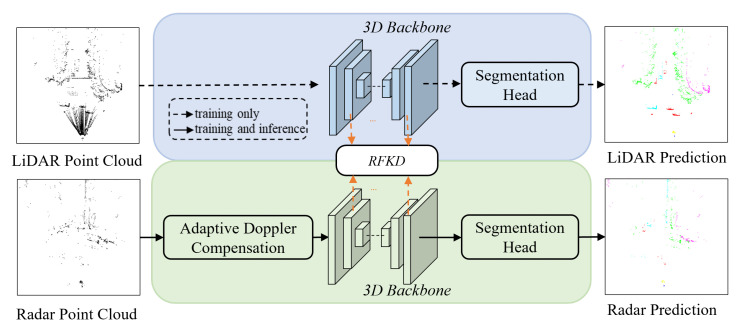
The framework of our RaSS model. Both the LiDAR (teacher) and the 4D radar (student) branch use the same backbones. The radar point cloud is pre-processed by the Adaptive Doppler Compensation (ADC) module to eliminate the speed produced by the ego motion. Radar Feature Knowledge Distillation (RFKD) module is designed to enhance the radar’s feature extraction at different scales during training. In inference, only the radar branch is involved.

The overview framework is depicted in Figure 4, where a LiDAR-based semantic segmentation model is employed as the teacher, and a radar-based model acts as the student. In the student branch, prior to feeding into the backbone, the radar point cloud gets preprocessed by an Adaptive Doppler Compensation module. In addition, during the training process, multi-scale feature distillation is performed between the LiDAR features and the Radar features. In inference, only the radar-based student model participates. For distillation facilitation, both teacher and student models utilize the same backbone. Next, we give more details about the Adaptive Doppler Compensation (ADC) and the Radar Feature Knowledge Distillation (RFKD) modules.

### 4.2. Adaptive Doppler Compensation (ADC)

**Figure 5:**
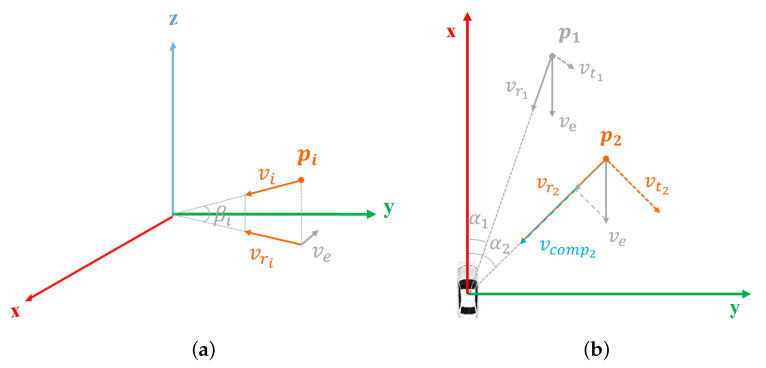
Illustration of the Adaptive Doppler Compensation. (**a**) For each radar point $p_{i}$, its *x*-*y* plane velocity can be obtained with $v_{r_{i}}=v_{i}\times \cos\beta_{i}$. (**b**) For the static point $p_{1}$, $v_{r_{1}}$ is caused by the velocity of ego-vehicle $v_{e}$. While for the moving point $p_{2}$, $v_{r_{2}}$ consists of $v_{e}$ and the point’s self-velocity $v_{{\mathrm{comp}}_{2}}$.

The Doppler speed provided by the radar can be valuable for classifying the points of moving objects. However, the raw Doppler speed obtained from the 4D radar is only the relative radial speed, which is non-zero for the points from static backgrounds when the ego-vehicle is moving. Based on the observation (Figure 3) that most of the radar points belong to the static categories, we propose an Adaptive Doppler Compensation (ADC) algorithm for the radar. The module can work in the situation of unknown ego-vehicle speed. The process is illustrated in Figure 5 and described as follows.

(1) For simplicity, we assume that the ego-vehicle moves only on the *x*-*y* plane and heads along the *x* direction. For each radar point $p_{i}=\left(x_{i},y_{i},z_{i},snr_{i},v_{i},\alpha_{i},\beta_{i}\right)\in R^{7}$ in *j*th frame, we project the raw Doppler $v_{i}$ onto the *x*-*y* plane, resulting in the projected speed $v_{r_{i}}=v_{i}\times cos\beta_{i}$.
(2) Project the $v_{r_{i}}$ to *x*-*axis* to obtain the relative speed $v_{x_{i}}$ along the vehicle’s motion direction, with $v_{x_{i}}=v_{r_{i}}/cos\alpha_{i}$.
(3) Assuming the majority of points in the scene are from the static background, we discretize the $v_{x_{i}}$ and define a function Max_Count to count the votes within the frame. The highest count is regarded as the estimated ego-vehicle velocity for the current *j*th frame, i.e., $v_{e_{j}}=Max\text{\_}Count\left({\left\{v_{x_{i}}\right\}}_{i=1}^{N_{j}}\right)$.
(4) For the radar point $p_{i}$ in the *j*th frame, the compensated velocity is obtained by $v_{comp_{i}}=v_{i}-v_{e_{j}}\times cos\alpha_{i}$. Finally, we use $v_{comp_{i}}$ instead of the original $v_{i}$ and feed it into the student network as the initial feature.

### 4.3. Radar Feature Knowledge Distillation (RFKD)

Through the convolution layers within the LiDAR and radar backbones (based on Cylinder3D), we can derive multi-level features for each input modality. Owing to the significant discrepancies in quantity and density between LiDAR and mm-Wave radar point cloud, effectively establishing feature correspondences and facilitating distillation constitutes a critical challenge. To address this issue, we first perform feature aggregation on the LiDAR and radar voxel features extracted by each backbone within the bird’s-eye-view (BEV) perspective. Guided by spatial relationships, we then select features from both aggregated LiDAR and radar BEV features, yielding two sets of aligned cross-modal features for subsequent feature distillation.

For each layer, given the LiDAR feature tensor $F_{L}\in R^{|P_{L}|\times C}$ and radar feature tensor $F_{R}\in R^{|P_{R}|\times C}$, their corresponding voxel coordinates ${\mathrm{Voxel}}_{L}\in R^{|P_{L}|\times 3}$ and ${\mathrm{Voxel}}_{R}\in R^{|P_{R}|\times 3}$ are obtained with all coordinate ranges falling within the interval defined by H×W×D. Here, $F_{L}={\left\{f_{i}^{L}\right\}}_{i=1}^{|P_{L}|}$ and $F_{R}={\left\{f_{j}^{R}\right\}}_{j=1}^{|P_{R}|}$ denote the LiDAR and radar feature sets, respectively; $P_{L}$ and $P_{R}$ represent the sets of LiDAR and radar point indices, with $|P_{L}|$ and $|P_{R}|$ indicating the number of points in each set; *C* denotes the channel dimension of the features, while *H*, *W*, and *D* represent the voxelization boundaries corresponding to maximum height, maximum width, and maximum depth, respectively.

Subsequently, we leverage the voxel coordinates ${\mathrm{Voxel}}_{L}$ and ${\mathrm{Voxel}}_{R}$ to perform feature aggregation within the bird’s-eye view (BEV) perspective, yielding the aggregated features $F_{L}^{P_{L}\to G_{L}}\in R^{|G_{L}|\times C}$ and $F_{R}^{P_{R}\to G_{R}}\in R^{|G_{R}|\times C}$, where $G_{L}$ and $G_{R}$ denote the sets of occupied BEV grid indices derived from the LiDAR point set $P_{L}$ and radar point set $P_{R}$, respectively; $|G_{L}|$ and $|G_{R}|$ indicate the number of non-empty grid cells after aggregation. Their corresponding grid coordinates ${\mathrm{Grid}}_{L}\in R^{|G_{L}|\times 2}$ and ${\mathrm{Grid}}_{R}\in R^{|G_{R}|\times 2}$ are obtained with each entry in ${\mathrm{Grid}}_{i}$ (i∈{L,R}) representing the 2D coordinates of the centroid of the corresponding occupied grid cell.

Given that the point cloud distributions of radar and LiDAR are different, we aim to perform knowledge distillation only in regions where both LiDAR and radar grids are occupied with points. To this end, guided by the spatial position of ${\mathrm{Grid}}_{L}$ and ${\mathrm{Grid}}_{R}$, we then select and obtain a set of matched features $F_{L}^{G_{L}\to G_{L}^{\prime }}\in R^{|G_{L}^{\prime }|\times C}$ and $F_{R}^{G_{R}\to G_{R}^{\prime }}\in R^{|G_{R}^{\prime }|\times C}$, where $G_{L}^{\prime }\subseteq G_{L}$ and $G_{R}^{\prime }\subseteq G_{R}$ denote the subsets of point-occupied and spatially matched grid indices, with $|G_{L}^{\prime }\left|=\right|G_{R}^{\prime }|$ indicating the number of successfully matched pairs.

Then, we pass the selected radar BEV feature $F_{R}^{G_{R}^{\prime }}\in R^{|G_{R}^{\prime }\times C}$ through a multi-layer perceptron (MLP) based adapter to reduce the domain gap prior to distillation. The adapted radar features are subsequently used to compute the distillation loss $L_{\mathrm{distill}}$ to the corresponding LiDAR features $F_{L}^{G_{L}^{\prime }}$, formulated in Equation (1):

(1) $$L_{\mathrm{distill}}=D\left(F_{L}^{G_{L}^{\prime }},\mathrm{MLP}\left(F_{R}^{G_{R}^{\prime }}\right)\right),$$

where D(·,·) specifically represents the mean squared error (MSE) metric.

**Figure 6:**
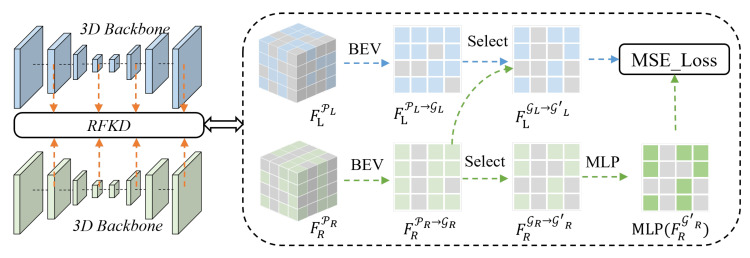
Illustration of the Radar Feature Knowledge Distillation (RFKD) module. For each layer, we separately aggregate the LiDAR and radar point features in the BEV view, apply the selection operation to both modality features, and then use a MLP-based adapter for radar features before calculating the MSE loss.

To ensure adequate learning of multi-scale feature extraction in the radar network, we distill features from deeper layers, which are organized according to the feature pyramid structure in the backbone. The details of the RFKD are shown in Figure 6.

### 4.4. Loss Functions

For the semantic segmentation task, we employ the Lovasz-Softmax loss [34] and Cross Entropy (CE) loss. Combined with the feature knowledge distillation loss $L_{\mathrm{distill}}$, the overall loss function is formulated as:

(2) $$L_{\mathrm{Total}}=L_{\mathrm{Seg}}+\frac{1}{K}\sum_{l=1}^{K}\lambda_{l}L_{\mathrm{distill}}^{\left(l\right)},$$

where $L_{\mathrm{Seg}}=L_{\mathrm{Lovasz}}+L_{\mathrm{CE}}$ denotes the segmentation loss, *K* represents the number of distillation layers, $\lambda_{l}$ denotes the corresponding weighting coefficient for each layer *l*, and $L_{\mathrm{distill}}^{\left(l\right)}$ is the distillation loss computed at layer *l* using Equation (1).

## 5. Experiments

### 5.1. Experiment Setups

#### 5.1.1. Dataset

We mainly evaluate the RaSS method on our proposed ZJUSSet. The annotated data are sequentially partitioned into training and validation subsets: the former comprises the first 3000 frames, and the latter contains the remaining 794 frames. For both subsets, we focus our evaluation on points within the range of [−5 m, 5 m] along the *z*-axis and [0, 100 m] along the *x*-axis. On the ZJUSSet dataset, we evaluate performance across 9 categories: building, vegetation, fence, car, cyclist, pedestrian, truck, bus and tricycle. After calibration, the reprojection error (RMSE) between the point clouds of mm-Wave radar and the LiDAR is 7.26 cm.

Meanwhile, we also use VoD dataset validate the generalizability of our algorithm. As VoD dataset ia originally designed for 4D mm-Wave radar 3D object detection, we generate pseudo-semantic segmentation labels for the annotated objects. Specifically, we leverage ground truth 3D object bounding box (bbox) for auxiliary annotation generation. Each point within the bbox is assigned the class label of the corresponding box, enabling point-level semantic annotation for six movable categories: car, pedestrian, cyclist, motor, truck, and bicycle. Finally, we filtered out frames with fewer than 100 annotated points, resulting in 2701 frames for training and 703 frames for validation. Although this is not a complete semantic segmentation dataset (with labeled points only accounting for approximately 10% of the total points), it provides complementary validation in more diverse scenarios.

#### 5.1.2. Evaluation Metrics

The performance is evaluated using the commonly adopted Intersection over Union (IoU), where the mean IoU (mIoU) across all categories is computed as:

(3) $$mIoU=\frac{1}{N}\sum_{i=1}^{N}\frac{TP_{i}}{TP_{i}+FP_{i}+FN_{i}}$$

where $TP_{i}$, $FP_{i}$, $FN_{i}$ donates True Positive, False Positive, and False Negative predictions for class *i*, and N=9 is the total number of categories.

For each category, we also use recall and precision to give a in-depth observation of each category, as shown in Equation (4) and Equation (5) respectively.

(4) $$Recall=\frac{TP}{TP+FN}$$

(5) $$Precison=\frac{TP}{TP+FP}$$

#### 5.1.3. Network Setups

We separately choose Cylinder3D [35] and PT-V2 [36] as the 3D backbones in our method. In each experiment, the same backbone is employed for the Teacher and the Student. The cylinder voxel size is configured as 1000 × 128 × 64, with each voxel corresponding to a spatial size of [0.1 m, 0.88°, 0.16 m]. For Cylinder3D, which consists of 8 feature layers in total, we apply the RFKD model to layers 2, 4, 6, and 8. While PT-V2, which has 9 feature layers, we select layers 1, 3, 6, and 8. Additionally, since PT-V2 does not include a voxelization step, we perform feature alignment between LiDAR and radar using K-Nearest Neighbors (KNN) with k=5.

#### 5.1.4. Training and Inference Details

Regarding the loss function, we configure the weights of the RFKD module as follows: $\lambda_{1}=1,\lambda_{2}=1,\lambda_{3}=0.1,\lambda_{4}=0.1$ for Cylinder3D, and $\lambda_{1}=\lambda_{2}=\lambda_{3}=\lambda_{4}=0.01$ for PT-V2. And we applied data augmentation to the training data, including rotating the point cloud within the range of [−22.5°, 22.5°], randomly flipping the point cloud along the y-axis, randomly scaling the point cloud with scales within [0.95, 1.05], along with the introduction of zero-mean Gaussian noise characterized by a standard deviation of 0.1. The model underwent training for 75 epochs, using a batch size of 32. We utilized the Adamw optimizer and OneCycleLR strategy to dynamically adjust the learning rate with a maximum learning rate of $2\times 10^{-4}$ for Cylinder3D and 0.02 for PT-V2. All the experiments were performed on a single Nvidia GeForce RTX 4090 GPU. The training time is 3 h for Cylinder3D and 2 h for PT-V2 backbones.

### 5.2. Experimental Results

#### 5.2.1. Results on ZJUSSet

**Figure 7:**
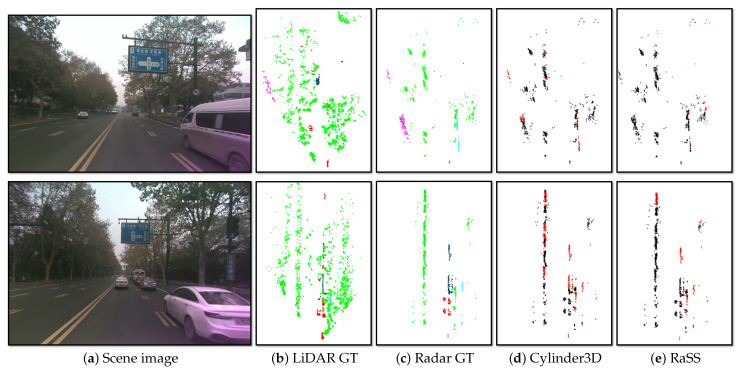
Qualitative results on the ZJUSSet validation set. The results are from the Cylinder3D-based RaSS (**a**). The meaning of colors in (**b**,**c**) are the same as in Figure 1c. For clarity, the correct and incorrect segmentation results are separately marked in black and red in (**d**,**e**).

Table 2 shows the semantic segmentation results on ZJUSSet. Firstly, our RaSS model ranks the top among all of the radar-input models and outperforms the baseline by 5.42% mIoU for Cylinder3D and 5.57% for PT-V2. It should mainly thank to the RFKD module, which can transfer the teacher’s feature extraction capability to the radar-based student model. Secondly, despite the highly sparse radar input, the RaSS model can still achieve 42.35% mIOU, exhibiting the surprising potential of 4D radar-based semantic segmentation for the autonomous vehicle. Lastly, the RaSS model still performs behind the LiDAR-based baseline model. This can be attributed to the sparser and noisier characteristics of mm-Wave radar point cloud. The qualitative results are presented in Figure 7.

**Table 2:** Semantic segmentation results on the ZJUSSet Validation set. L and R separately stand for LiDAR and mm-Wave radar data. RaSS(C) and RaSS(P) represents RaSS using Cylinder3D or PT-V2 as backbone, respectively. The bold and underlined values denote the best and second place within the radar input methods, respectively.

Method	Input	mIoU	Building	Fence	Vegetation	Car	Cyclist	Pedestrian	Truck	Bus	Tricycle
Cylinder3D [35]	L	69.02	72.36	63.17	91.95	95.52	69.39	68.30	44.41	84.71	31.33
PT-V2 [36]	L	72.47	80.46	62.45	93.60	96.66	79.65	77.34	48.66	84.46	28.95
KPConv [18]	R	29.20	55.70	55.90	42.90	68.40	8.30	15.40	3.20	12.90	0.10
PolarNet [37]	R	31.57	71.56	46.08	57.04	73.41	1.73	0.68	0.09	33.49	0.01
Minkowski [38]	R	33.42	61.06	58.56	51.92	75.10	7.13	15.73	2.81	25.25	**3.19**
Cylinder3D [35]	R	36.15	73.82	58.61	58.64	69.35	5.62	29.75	3.11	26.34	0.14
PT-V2 [36]	R	36.78	**75.53**	62.32	**63.81**	62.07	3.69	18.56	4.76	40.05	0.25
RaSS(C)	R	41.57	73.86	63.3	61.08	**75.83**	**16.20**	34.30	**9.64**	38.82	1.11
RaSS(P)	R	**42.35**	74.09	**63.46**	61.99	69.06	9.66	**51.74**	7.82	**42.37**	0.94

Considering the practical application of semantic segmentation in autonomous driving, we statistically analyzed the network’s real-time performance and memory footprint. Experimental results demonstrate that our model fully meets real-time requirements (74 ms with Cylinder3D, 60 ms with PT-V2) and is faster than LiDAR-based counterparts (79 ms and 66 ms for LiDAR with the same backbone, respectively). Moreover, radar exhibits significantly lower memory usage than LiDAR (1.5 GB versus 6.3 GB with Cylinder3D; 0.4 GB versus 1.1 GB with PT-V2).

Table 3 presents the IoU, precision, and recall for each category in the ZJUSSet. For categories with few points, Pedestrian achieves higher precision and recall than Cyclist, Truck, and Tricycle, possibly because more training samples are available in the training set.

#### 5.2.2. Results on VoD Dataset

We validated our method on the self-constructed VoD dataset and the results are shown in Table 4. With less categories for segmentation, the mIoU becomes higher than those in the ZJUSSet. Our method still outperforms the baselines with a noticeable margin.

### 5.3. Failure Cases Analysis

**Figure 8:**
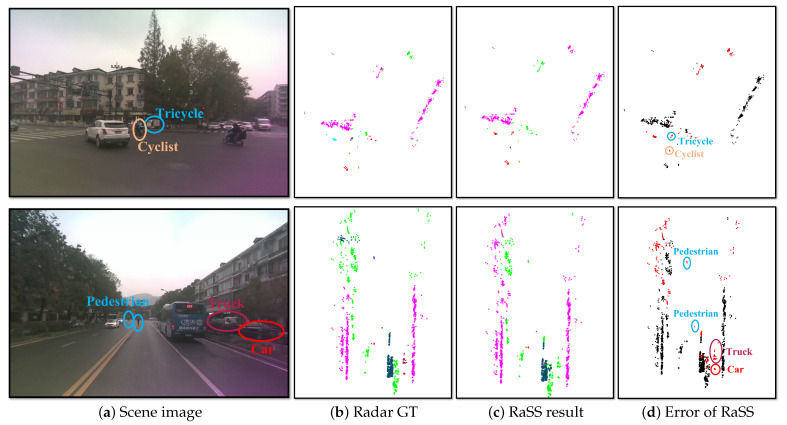
Failure cases on the ZJUSSet validation set. The meaning of colors in (**b**,**c**) are the same as in Figure 1c. For clarity, the correct and incorrect segmentation results are separately marked in black and red in (**d**). Different colored circles in (**a**,**d**) denote distinct categories.

Some failure cases on the ZJUSSet validation set are presented in Figure 8. In the top of the Figure 8, the model shows poor segmentation performance on the minority categories, with IoUs as follows: cyclist (10.45%) and tricycle (0%). The bottom of Figure 8 illustrates another failure case, where the model produces false semantic labels for some minority categories (truck), small object (pedestrian), and the categories with sparse point cloud (in this case, car). The detailed IoUs are: truck (0%), pedestrian (0%) and car (16.67%).

### 5.4. Ablation Study

Ablation studies were conducted to validate the effectiveness of key components in our framework. The results are summarized in Table 5.

We first evaluated the impact of the ADC module for motion compensation. As shown in the rows 1–2 of the Table 5, enabling the ADC module improved mIoU by 2.3% compared to the baseline. This confirms that accurate motion alignment is crucial for enhancing the representation capability of radar features.

We also compared different knowledge distillation strategies in the rows 3–4, 6 of the Table 5. Logits-based distillation and feature-based distillation each improved mIoU by 1.07% and 4.18%, respectively, when applied individually. However, their combination not only increased learning complexity but also resulted in a performance drop, with an mIoU that is 0.99% lower than that of feature-based distillation alone. This indicates significant redundancy or potential conflict between the two distillation mechanisms, underscoring the need to avoid arbitrary combination of distillation strategies and instead prioritize the selection of a single effective mechanism.

Regarding the number of distillation layers, a trade-off exists between knowledge quantity and transfer efficiency. As shown in the last 3 rows of the Table 5, increasing the number of distillation layers from 2 to 4 improved mIoU by 3.21%, but further increasing to 8 layers led to a 1.8% drop. This suggests that excessive layers may introduce noisy or redundant information, underscoring the need for optimal layer selection in knowledge distillation.

Finally, we evaluated the functionality of the feature selection operation for LiDAR and radar features described in Section 4.3. As detailed in Section 4.3, we perform selection on the BEV features of both LiDAR and radar based on spatial correspondence between ${\mathrm{Grid}}_{L}$ and ${\mathrm{Grid}}_{R}$, which effectively restricts knowledge distillation to grid regions commonly occupied by both modalities. In contrast, the approach only aligns LiDAR features using the same spatial correspondence criteria, thereby performing distillation across all radar-occupied grid regions. Experimental results in the rows 5–6 of the Table 5 demonstrate the effectiveness of our proposed strategy: despite the richer and more discriminative nature of LiDAR features, enforcing alignment between all radar features and their corresponding LiDAR features leads to performance degradation.

## 6. Conclusions and Future Works

Semantic segmentation for 4D mm-Wave radar remains an unexplored and attractive domain in robotics and autonomous driving field. In this paper, we presented a new dataset for 4D radar point cloud semantic segmentation, namely ZJUSSet, and proposed a knowledge distillation-based method to fulfill this task. The ZJUSSet provides data from 4D radar, LiDAR, and camera, and annotates the point cloud with up to 10 categories. To mitigate the difficulties stemming from the sparsity and noise, we introduce a cross-modal distillation mechanism for semantic segmentation. It encourages the radar model to extract similar features like LiDAR, to improve the performance. We also design the Doppler compensation module to provide more explicit motion information for the points. Thanks to all these delicate designs, our PT-V2 based RaSS version achieves 5.57% mIOU improvements over the baseline on ZJUSSet. While there still remains a performance gap compared to LiDAR, this undoubtedly marks a promising start, which will hopefully inspire more research into exploring more potential of 4D mm-wave radar. In our future work, we plan to augment the ZJUSSet with more data under adverse weathers, and develop more advanced algorithms to improve the semantic segmentation performance.

## Figures and Tables

**Table 1:** The semantic segmentation datasets containing mm-Wave radar data.

Dataset	Year	Radar	Camera	LiDAR	Annotation	Categories
CARRADA [7]	2020	3D	RGB		2D Box, 2D Pixel	4
RADIal [8]	2021	3D	RGB	16-line	2D Box, 2D point	1
RadarScenes [9]	2021	3D	RGB		2D Point	6
ZJUSSet (**Ours**)	2024	4D	RGB	Livox (∼128-line)	3D Point	10

**Table 3:** IoU, Precision, and Recall Metrics for Each Category in the Validation set of ZJUSSet. The results are from the PT-V2-based RaSS.

Metric	Building	Fence	Vegetation	Car	Cyclist	Pedestrian	Truck	Bus	Tricycle
IoU	74.09	63.46	61.99	69.06	9.66	51.74	7.82	42.37	0.94
Precision	79.20	76.82	80.90	80.95	13.44	85.79	21.56	79.10	2.57
Recall	91.99	78.49	72.62	82.46	25.57	56.59	10.93	47.71	1.46

**Table 4:** Semantic segmentation results with pseudo-labels on the VoD Validation set. L and R separately stand for LiDAR and mm-Wave radar data. RaSS(C) and RaSS(P) represents RaSS using Cylinder3D and PT-V2 as backbone, respectively. The bold and underlined values denote the best and second place within radar input methods, respectively.

Methods	Input	mIoU	Car	Pedestrian	Cyclist	Motor	Truck	Bicycle
Cylinder3D	L	74.82	89.93	87.19	79.62	61.51	43.80	86.89
PTV2	L	74.71	77.09	89.81	83.51	87.68	21.12	89.05
Cylinder3D	R	61.22	**87.81**	71.97	77.02	1.50	71.10	**57.92**
PTV2	R	62.65	85.49	72.49	80.91	0.00	84.55	52.44
RaSS(C)	R	**65.73**	87.63	**75.17**	**82.17**	6.64	**86.64**	56.15
RaSS(P)	R	64.49	82.40	69.26	78.67	**38.81**	65.08	52.71

**Table 5:** Performance comparison of different configurations on ZJUSSet. Herein, both the baseline and RaSS employ Cylinder3D as their backbone. ADC stands for the Adaptive Doppler Compensation. K represents the number of distillation layers adopted. Selected (marked with ) refers to the Selected feature distillation described in Section 4.3, whereas an empty entry denotes distillation for all non-empty radar grids, regardless of whether there are corresponding non-empty LiDAR features at that location.

Network	ADC	Logits-KD	Feats-KD	K	Selected	mIoU
Baseline				0		36.15
RaSS	✓			0		37.39
	✓	✓		0		38.46
	✓	✓	✓	4	✓	40.58
	✓		✓	4		40.29
	✓		✓	4	✓	**41.57**
	✓		✓	2	✓	38.36
	✓		✓	8	✓	39.77

## Data Availability

The original contributions presented in this study are included in the article. Further inquiries can be directed to the corresponding author.

## Abbreviations

The following abbreviations are used in this manuscript:

mm-Wave	millimeter-wave [34]
PT-V2	PointTransformer-V2
RaSS	Radar Point Cloud Semantic Segmentation
VoD	The View of Delft dataset
ADC	Adaptive Doppler Compensation
RFKD	Radar Feature Knowledge Distillation
BEV	bird’s-eye-view
